# A six-minute step test protocol for the investigation of dyspnea

**DOI:** 10.1590/S1806-37132014000600012

**Published:** 2014

**Authors:** Aline Aparecida Simsic, Ada Clarice Gastaldi, José Baddini-Martinez

**Affiliations:** University of São Paulo, Ribeirão Preto School of Medicine, Ribeirão Preto, Brazil, University of São Paulo at Ribeirão Preto School of Medicine, Ribeirão Preto, Brazil; University of São Paulo, Ribeirão Preto School of Medicine, Ribeirão Preto, Brazil, Graduate Program in Clinical Medicine, University of São Paulo at Ribeirão Preto School of Medicine, Physical Therapy Course, Ribeirão Preto, Brazil; University of São Paulo, Ribeirão Preto School of Medicine, Ribeirão Preto, Brazil, University of São Paulo at Ribeirão Preto School of Medicine, Ribeirão Preto, Brazil

## To the Editor:

Dyspnea is a common symptom and a major contributor to poor quality of life in respiratory disease.^(1,2) ^Although the ideal approach to alleviate dyspnea is the treatment of its primary etiology, there are situations in which this symptom persists regardless of the best available therapies.

Since no highly effective pharmacological agents against dyspnea per se are currently available, the search for new drugs should be a priority.^(^ 1 ^)^ Studies aimed at investigating the physiological aspects of dyspnea usually demand complex methodology, including central nervous system imaging and cardiopulmonary exercise tests. Although these methods will always be necessary to characterize mechanisms of action and physiological effects of new anti-dyspnea drugs, the selection of promising agents might be made faster and less expensive with the use of simpler methodologies.

Step tests have been used with healthy subjects, and many protocols have been adapted for those with cardiopulmonary disease.^(3-5) ^These tests require little space and are suitable to obtaining continuous cardiac and symptom monitoring in a simple way. 

We have developed a six-minute step test (6MST) protocol aiming at obtaining consistent and reproducible dyspnea responses in patients with COPD. The institutional review board approved the study. We included 16 patients (14 men; mean age = 64.3 ± 8.1 years; FEV_1 _= 37.6 ± 14.5%; FEV_1_/FVC = 42.4 ± 10.3%; oxyhemoglobin saturation = 94.9 ± 2.2%), who made three visits to the laboratory. The mean time intervals between visits 1 and 2 and between visits 2 and 3 were, respectively, 4.8 ± 3.1 days and 5.5 ± 2.1 days. The subjects stepped up on and down from a 20-cm-high wooden bench in a self-paced rhythm for 6 min. Monitoring included pulse oximetry and dyspnea scores (employing a numeric scale ranging from 0 to 10) every 2 min. The volunteers performed two tests in the initial visit, 30 min apart. The highest number of steps climbed in that visit was employed for calculating the speed to be applied in additional evaluations. A computer-based, sound emitting electronic metronome paced the rhythm of the other tests. The patients randomly received either inhaled placebo or an inhaled dose of albuterol (200 mg) 20 min prior to the 6MSTs in visits 2 and 3. Because the scores of dyspnea at rest exhibited some variation, corrections were made by subtracting the scores at 2, 4, 6 and 8 min from the baseline value.

**Figure 1 -:**
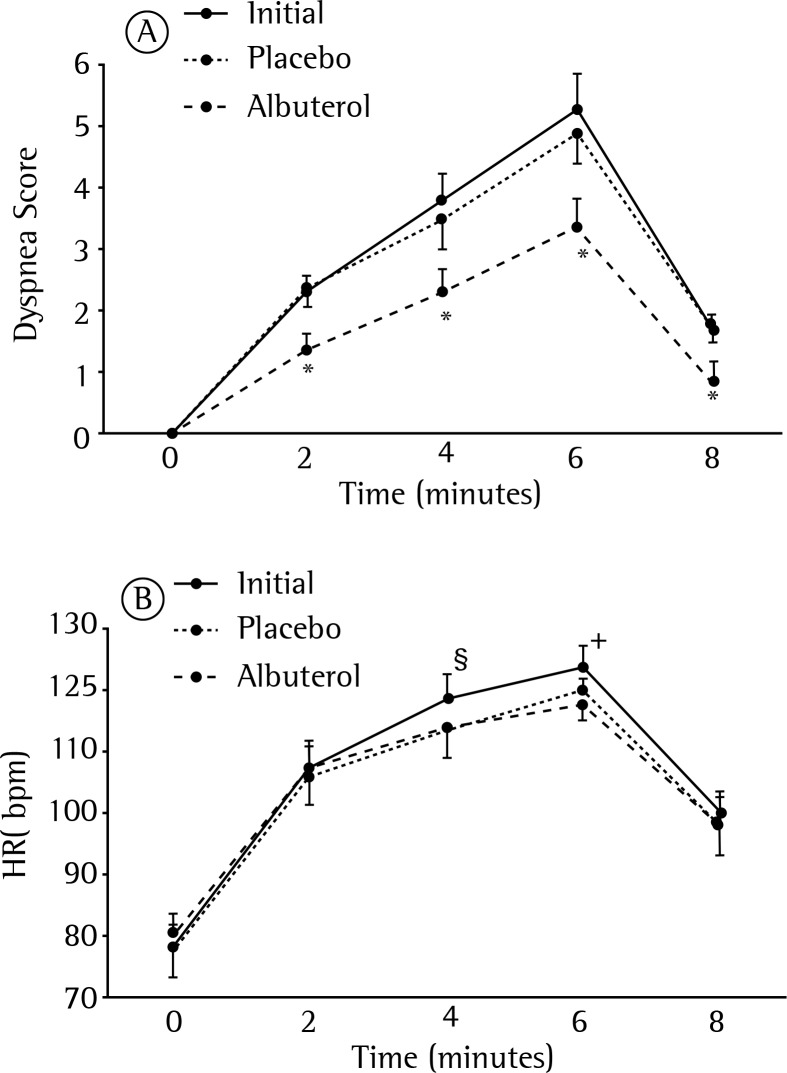
Results of the 6MST protocol for the investigation of dyspnea obtained from 16 stable COPD patients. In A, dyspnea scores In B, HR in bpm. *§p < 0.05 initial test vs. use of placebo. +p < 0.05 initial test vs. use of albuterol. ANOVA and Tukey's post hoc test.

Dyspnea scores were similar in visit 1 and after the use of inhaled placebo (Figure 1). Albuterol significantly decreased the dyspnea scores during exercise. There were no significant differences in the comparisons between HRs measured after the use of placebo and albuterol. However, HRs in visit 1 6MST were higher than were those observed after the use of placebo at 4 min and after the use of albuterol at 6 min. The last findings may be explained by the self-paced trait of the initial test. The use of a metronome during visits 2 and 3 most probably led to a more homogeneous distribution of the efforts throughout the test.

The 6MST protocol for the investigation of dyspnea was easily performed and well tolerated by the patients. The procedures are simple, are inexpensive, and induce dyspnea in a reproducible fashion. In addition, this method allows the introduction of additional monitoring, such as ventilatory parameters and oxygen consumption measurements, if necessary. The present protocol has the potential to become a useful tool for investigating anti-dyspnea interventions in clinical practice.
